# Gradual Increase of High Mobility Group Protein B1 in the Lungs after the Onset of Acute Exacerbation of Idiopathic Pulmonary Fibrosis

**DOI:** 10.1155/2011/916486

**Published:** 2011-02-21

**Authors:** Masahito Ebina, Hiroyuki Taniguchi, Taku Miyasho, Shingo Yamada, Naoko Shibata, Hiromitsu Ohta, Shu Hisata, Shinya Ohkouchi, Tsutomu Tamada, Hidekazu Nishimura, Akitoshi Ishizaka, Ikuro Maruyama, Yoshinori Okada, Kondo Takashi, Toshihiro Nukiwa

**Affiliations:** ^1^Department of Respiratory Medicine, Tohoku University Graduate School of Medicine, 1-1 Seiryo, Aoba-ku, Sendai 980-8574, Japan; ^2^Department of Respiratory Medicine, Tosei General Hospital, Seto, Aichi 489-8642, Japan; ^3^Department of Veterinary Biochemistry, Rakuno Gakuen University, Ebetsu, Hokkaido 069-8501, Japan; ^4^Central Institute, Shino-Test Corporation, Kanagawa 229-0011, Japan; ^5^Virus Research Center, National Hospital Organization, Sendai 983-0045, Japan; ^6^Department of Medicine, Keio University, Tokyo 160-8582, Japan; ^7^Department of Laboratory and Molecular Medicine, Kagoshima University, Kagoshima 890-8520, Japan; ^8^Department of Thoracic Surgery, Tohoku University Hospital, Sendai 980-8574, Japan

## Abstract

The pathogenesis of acute exacerbation of idiopathic pulmonary fibrosis (IPF) remains to be elucidated. To evaluate the roles of inflammatory mediators in acute exacerbation, the concentrations of high mobility group protein B1 (HMGB1), a chief mediator of acute lung injury, and 18 inflammatory cytokines were measured in the bronchoalveolar lavage fluid, serially sampled from seven IPF patients after the onset of acute exacerbation. HMGB1 gradually increased in the alveolar fluid after the onset of acute exacerbation, in positive correlation with monocytes chemotactic protein-1 (MCP-1), a potent fibrogenic mediator. In the lung tissues of eight IPF patients autopsied after acute exacerbation, intense cytoplasmic staining for HMGB1 was observed in the alveolar epithelial cells in alveolar capillary augmented lesions, where the capillary endothelial cells remarkably reduced the expression of thrombomodulin, an intrinsic antagonist of HMGB1. These results suggest pathogenic roles for HMGB1 and MCP-1 in the late phase of acute exacerbation of IPF.

## 1. Introduction

Idiopathic pulmonary fibrosis (IPF) is one of the most refractory of all lung diseases lacking effective therapy [1]. Not only progressive pulmonary fibrosis but also acute exacerbation, defined as clinically significant deterioration of unidentifiable cause, is attributed to the poor survival of these patients [2]. In the pathologic findings in the lungs of IPF patients afflicted with acute exacerbation, diffuse alveolar damage, which is consistent with the findings observed after acute lung injury from various causes [2], is the most characteristic underlying usual interstitial pneumonia. However, the etiology of acute exacerbation of IPF has yet to be elucidated.

High mobility group protein B1 (HMGB1) was originally identified as a nuclear nonhistone protein with DNA-binding domains and implicated as an important endogenous danger signaling molecule [3] as well as a late mediator of systemic inflammation in septic shock [4, 5], thus having a putative role in the pathogenesis of acute lung injury [6, 7]. In addition, several studies have identified the B-box domain of HMGB1 as important for many of the proinflammatory properties of HMGB1, including cytokine release [8, 9]. HMGB1 is released passively during cellular necrosis by almost all cells which have a nucleus and signals to neighboring cells in the case of ongoing damage [5]. However, HMGB1 also is secreted actively by immune cells such as monocytes, macrophages, and dendritic cells [4, 10, 11]. The receptor for advanced glycation end products (RAGE) was the first receptor demonstrated to bind HMGB1 [12], and HMGB1 signaling through RAGE was found to promote chemotaxis and the production of cytokines in a process that involves the activation of the transcription factor nuclear factor-*κ*B (NF-*κ*B) [13, 14]. Nevertheless, the contribution of HMGB1 in the pathogenesis of acute exacerbation of IPF has not been determined yet.

We previously reported the heterogeneous remodeling of CD34-positive alveolar capillaries in the lungs of patients with IPF [15]. The alveolar capillaries were decreased in fibrotic lesions, but increased in nonfibrotic alveolar septa around the fibrotic lesions, where VEGF and IL-8, potent angiogenic factors, were augmented [15]. Since VEGF is also a potent inducer of vascular permeability [16], these capillary dense lesions in nonfibrotic alveolar septa are considered to be leaky, and thus susceptible to the alveolar infiltration triggered by inflammatory mediators in acute lung injury [17]. We are also interested in thrombomodulin, an endothelial anticoagulant cofactor, which is highly expressed in alveolar capillaries in normal control lungs but less so in IPF lungs [15]. This is because the N-terminal domain of thrombomodulin binds HMGB1 so as to prevent its interaction with RAGE and thus suppresses the induction of proinflammatory events [18].

In this context, we examined the bronchoalveolar lavage fluid serially sampled from patients with IPF after acute exacerbation, along with the lung tissue specimens, biopsied from patients with stable IPF and autopsied from IPF patients who died after acute exacerbation, to evaluate the involvement of inflammatory mediators in the pathogenesis of acute exacerbation of IPF. 

## 2. Materials and Methods

**Clinical Samples**

Acute exacerbation of idiopathic pulmonary fibrosis was diagnosed according to the Japanese diagnostic criteria established in 2004 [19], which are essentially compatible with the criteria by the Idiopathic Pulmonary Fibrosis Clinical Research Network Investigators in 2007 [2]. Bronchoalveolar lavage fluid was sampled at Tosei General Hospital from seven patients with IPF after two to four instances of acute exacerbation in each patient to allow for the diagnostic exclusion of infection, and the samples were stored at −80°C degrees. The surgical biopsies of ten patients with stable IPF, and the autopsied lungs of eight patients with IPF who died after acute exacerbation, were obtained at Tohoku University Hospital. The disease-free lung tissues autopsied from three patients without any chronic lung diseases were also examined as controls. These lung tissues were fixed in 10% buffered formalin and imbedded in paraffin wax. The written informed consent obtained from the patients for using these clinical samples was approved by the ethics committee of Tohoku University.

**Measurement of HMGB1 and Cytokines**

HMGB1 in BALF was measured by a highly sensitive and specific ELISA method (Shino-test, Kanagawa, Japan) [20], as reported previously [6, 7]. In brief, black polystyrene microtiter plates (Corning Laboratory Science, Corning, NY) were coated with anti-HMGB1 polyclonal antibody in phosphate-buffered saline (PBS), washed 3 times with PBS containing 0.05% Tween-20, and blocked by PBS-1% bovine serum albumin for 2 hours. After washing, 100 *μ*l of 8 dilutions each of the standard and samples, 1 : 1 dilutions in 0.2 mol/L Tris (pH 6.5), 0.15 mol/L NaCl containing 1% BSA were added to the wells and incubated for 24 hours at room temperature. After washing, antihuman HMGB1 peroxidase-conjugated monoclonal antibody was added and incubated at room temperature for 30 minutes. After another washing step, PS-atto (Lumigen, Southfield, MI) was added, and its luminescence was measured with a 9000D microplate luminescence reader (DIA-IATRON, Tokyo, Japan). The concentrations of 17 cytokines in these BALF samples, consisting of Interleukin (IL)-1*β*, IL-2, IL-4, IL-5, IL-6, IL-7, IL-8, IL-10, IL-13, IL-17, granulocyte colony stimulating factor (G-CSF), monocytes chemotactic protein-1 (MCP-1), granulocyte/macrophage-colony stimulating factor (GM-CSF), macrophage inflammatory protein (MIP)-1*β*, interferon (IFN)-*γ*, and tumor necrosis factor (TNF-*α*), were determined using Bio-Plex (Bio-Rad Laboratories Inc.) following the manufacturer's protocol. The total TGF-*β*1 in these BALF samples was also measured using the enzyme immunoassay (EIA) produced by MBC Laboratories, Inc. (Tokyo, Japan).

**Virus Isolation**

Each BALF sample was subjected to virus isolation by the microplate method [21] at the Virus Research Center at the National Hospital Organization (Sendai, JAPAN). In short, the specimen was placed in transport medium composed of Eagle's minimum essential medium (Sigma, St. Louis, MO, USA) with 0.5% gelatin containing 500 units/ml of penicillin G and 500 mg/ml of streptomycin and was centrifuged at 4,000 g at 4°C for 15 min. The supernatant fraction was inoculated into cultured cells of human embryonic fibroblasts, using the cell lines, HEp-2; Vero; HMV-II, MDCK, LLC-MK2 cells, which can support the growth of numerous viruses, including respiratory syncytial virus, influenza viruses, parainfluenza viruses, enteroviruses, adenoviruses, cytomegalovirus, herpes simplex virus, rhinovirus, mumps virus, measles virus, human coronavirus 229E, and human metapneumovirus. After inoculation, cytopathic effects were examined daily for two weeks, and, furthermore, when no CPE was found, blind passage was performed once for another two weeks with the same system. RT-PCR was also performed for human metapneumovirus virus isolation [22].

**Immunohistochemistry**

Tissue sections of 3 *μ*m thickness were deparaffinized and treated with a peroxidase-blocking reagent (DAKO, Carpinteria, CA) for 10 minutes at room temperature before incubation with the primary antibodies. The antibodies and the optimal dilutions used in this study were as follows: monoclonal antibodies against human HMGB1 (1 : 3,000, Shino-test), the same antibody used for ELISA, RAGE (MAB5328, 1 : 50, CHEMICON International, Inc., Temecula, CA), thrombomodulin (TM1009, 1 : 100, DAKO Company Ltd., Glostrup, Denmark), CD34 (4A1, 1 : 100, Nichirei Co., Tokyo, Japan), and antihuman von Willebrand factor (vWF) monoclonal antibody (F8/86, 1 : 1,000, Nichirei). The tissues were incubated with a primary antibody in a moist chamber at 4°C for overnight and were further reacted with a polymer reagent (ENVISON kit; DAKO, Carpinteria, CA) for 60 minutes at room temperature. For double immunohistochemical staining, the antigen-antibody complex was visualized with Vector Red (Vector Laboratories, Burlingame, CA) or 1 mM 3.3′-diaminobenzidine (DAB), as described previously [15].

**Morphometric Evaluation**

The percentages of the distribution area of the cells immunoreactive for thrombomodulin among those immunoreactive for CD34 were estimated from 30 randomly selected color images at a magnification of 400 in each case using a digital image analysis system (Lumina Vision, Mitani Corporation, Fukui, Japan).

**Statistical Analysis**

For analysis of two unpaired samples, the nonparametric Mann-Whitney *U* test was used. A significant difference was defined as *P* < .05. All values were represented as the means ± SEM.

## 3. Results

### 3.1. Cytokines in BALF after Acute Exacerbation

**Figure 1:**
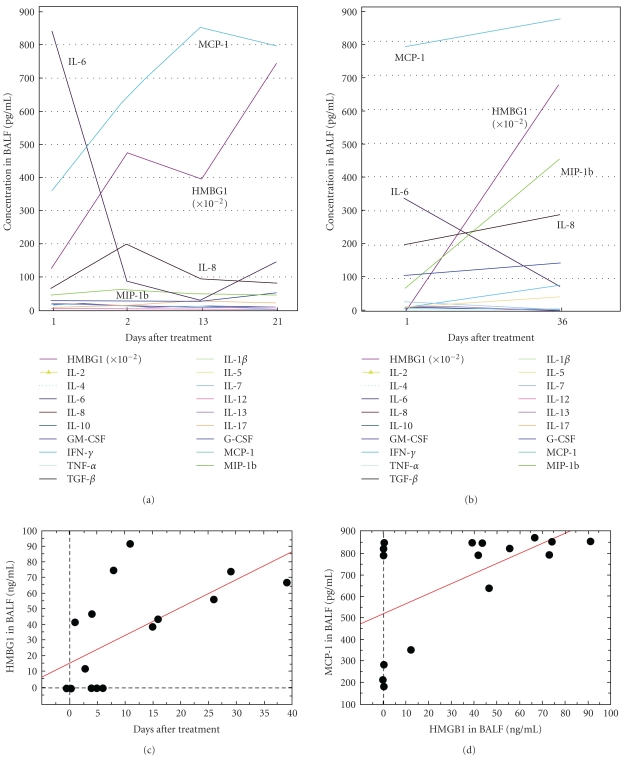
Inflammatory mediators in the BALF of IPF patients after acute exacerbation. (a) The concentrations of inflammatory mediators in the BALF of a 69-year-old male (an ex-smoker, Brickman index = 400) on days 1, 2, 13, and 21 after steroid therapy against acute exacerbation. He had been followed up without immunosuppressive therapy against IPF before administration to the hospital. No mechanical ventilator was used for treatment. He died 5 months later. (b) A 67-year-old female (a nonsmoker, no previous treatment for IPF) diagnosed with acute exacerbation 3 days after “flu-like” phenomena and died 3 months later. No mechanical ventilator was used for treatment. (c) The pooled data of the HMGB1 concentration in 16 BALF samples obtained from 7 patients showed a gradual increase of HMGB1 even after the steroid therapy for acute exacerbation (*r* = 0.64, *P* = .008). (d) Among the 18 cytokines in the BALF samples examined, only MCP-1 was increased in positive correlation with HMGB1 (*r* = 0.58, *P* = .0176) after the steroid therapy for acute exacerbation.

The bronchoalveolar lavage fluid (BALF) that we examined in this study was serially sampled two to four times after acute exacerbation from each IPF patient (*n* = 8). Although all of these patients were treated with steroid therapy after the diagnosis of acute exacerbation (Day 1), none of these patients were mechanically ventilated during the treatment. We measured the concentrations of HMGB1 and 18 other inflammatory cytokines, consisting of IL-1*β*, IL-2, IL-4, IL-5, IL-6, IL-7, IL-8, IL-10, IL-13, IL-17, G-CSF, MCP-1, GM-CSF, MIP-1*β*, IFN-*γ*, TNF-*α*, and TGF−*β*. The findings in two of these seven patients are illustrated (Figures 1(a) and 1(b)). Although the concentration of HMGB1 in BALF was low in the early phase of acute exacerbation, HMGB1, and MCP-1 as well, continued increasing even after steroid therapy. The pooled data (16 samples from 7 patients) of the HMGB1 concentration in the BALF of these seven patients also revealed a gradual increase of HMGB1 after acute exacerbation (Figure 1(c)). Among the inflammatory cytokines examined in these BALF samples, only MCP-1 was increased in positive correlation with HMGB1 (Figures 1(a) and 1(d)).

Our attempts at virus isolation in the BALF specimens sampled after acute exacerbation did not uncover any of the usual pathogenic bacteria or viruses, such as respiratory syncytial virus, influenza viruses, parainfluenza viruses, enteroviruses, adenoviruses, rhinovirus, cytomegalovirus, herpes simplex virus, coronavirus, mumps virus, or metapneumovirus (data not shown). 

### 3.2. HMGB1 Producing Cells in Lung Tissue

**Figure 2:**
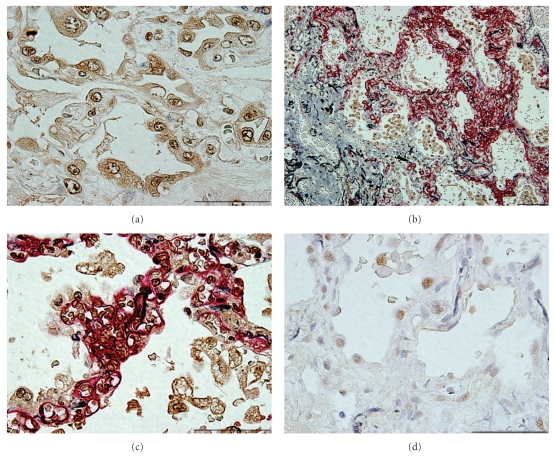
Immunohistochemical HMGB1 expression in lung tissues. (a) Intense cytoplasmic and nuclear staining HMGB1 was observed in alveolar macrophages and alveolar epithelial cells in the lungs of patients with IPF who died after acute exacerbation (HMGB1 in brown, bar = 50 *μ*m). (b) and (c) The immunoreactive cells for HMGB1 were distributed in the alveolar damaged lesions in which the alveolar capillaries were increased (CD34 in red and HMGB1 in brown; scale bars: 100 *μ*m in (b) and 50 *μ*m in (c)). (d) In the surgical lung biopsies of the IPF patients (d) revealed only nuclear staining for HMGB1 (HMGB1 in brown; scale bar: 50 *μ*m). The sections were counterstained with Elastica-Goldner staining, showing the elastic fibers in purple and collagen in light blue ((b)–(d)).

The immunohistochemical expression of HMGB1 in the lung tissues of 18 patients with IPF was compared between two patient groups: the autopsied lung tissues of eight patients who had who died after acute exacerbation and the surgically biopsied lung tissues of ten patients with stable IPF. In the autopsied lung tissues, intense cytoplasmic staining for HMGB1 was observed in most of alveolar macrophages and alveolar epithelial cells with or without nuclear staining (Figure 2(a)). Double immunostaining for HMGB1 and CD34, a marker of alveolar capillary endothelial cells [15], clearly revealed the distribution of these cells, with an intense expression of HMGB1 near the capillary augmented alveolar septa that were without apparent fibrosis (Figures 2(b) and 2(c)). In contrast, only nuclear staining for HMGB1 was observed in the surgical lung biopsies of the patients with stable IPF (Figure 2(d)).

**Figure 3:**
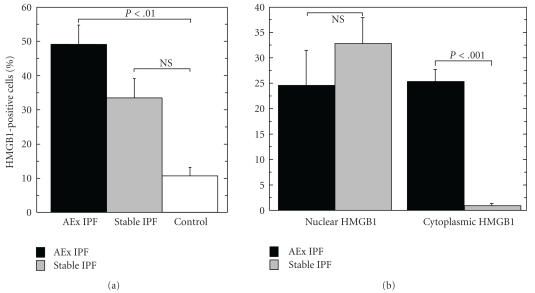
Quantitative distribution of HMGB1-positive cells in lung tissues. (a) The distribution patterns of the cells positive for HMGB1 were quantified in the autopsied lung tissues after acute exacerbation in 8 IPF patients (AEx IPF), using the biopsied lung tissues of 10 patients with stable IPF and three controls. (b) The percentages of immunoreactive cells with nuclear or cytoplasmic staining for HMGB1 are shown. The cells with both cytoplasmic and nuclear staining for HMGB1 were counted as cytoplasmic.

The average percentage of the cells immunoreactive for HMGB1 was 48.9 ± 2.51% (mean ± SEM) in the autopsied lung tissues of IPF patients after acute exacerbation (Figure 3). In the surgical biopsies of patients with stable IPF, 33.3 ± 5.6% of the cells were positive for HMGB1 without a significant difference compared with the autopsies (*P* = .057); however, most of these cells with HMGB1 immunoreactivity in the case of the surgical biopsies exhibited nuclear staining (Figure 2(d)) along with rare cytoplasmic staining (2.19 ± 0.46%), which was significantly different compared with the percentage of nuclear staining in autopsies (24.5 ± 7.0%, *P* < .001) (Figure 3). 

### 3.3. Expression of MCP-1, RAGE, and Thrombomodulin

**Figure 4:**
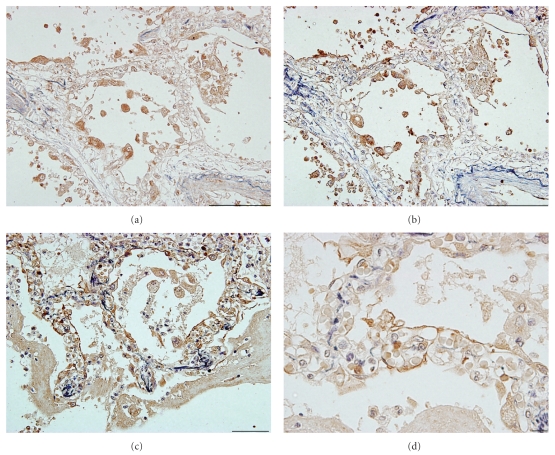
Distribution of HMGB1, MCP1 and RAGE in the lungs after acute exacerbation. The cells immunoreactive for HMGB1 (a) and MCP-1 (b) in the consecutive sections of the autopsied lung tissues of IPF patients who died after acute exacerbation (bars = 100 *μ*m). The intense immunoreactivity of RAGE, the receptor of HMGB1, was observed in type I alveolar epithelial cells in these lungs ((c) and (d)) ((c), bar = 100 *μ*m; (d), bar = 10 *μ*m). The sections were counterstained with Elastica-Goldner staining, showing the elastic fibers in purple and collagen in light blue ((a)–(d)).

The distribution of the cells immunoreactive for HMGB1 was compared with that of MCP-1 and RAGE, one of the main receptors for HMGB1, in the postmortem lung tissues of patients with IPF and acute exacerbation. The cells producing MCP-1 coincided with the cells having cytoplasmic HMGB1 expression (Figures 4(a) and 4(b)), while the intense expression of RAGE was chiefly observed in epithelial cells and to a lesser extent in capillary endothelial cells (Figures 4(c) and 4(d)).

**Figure 5:**
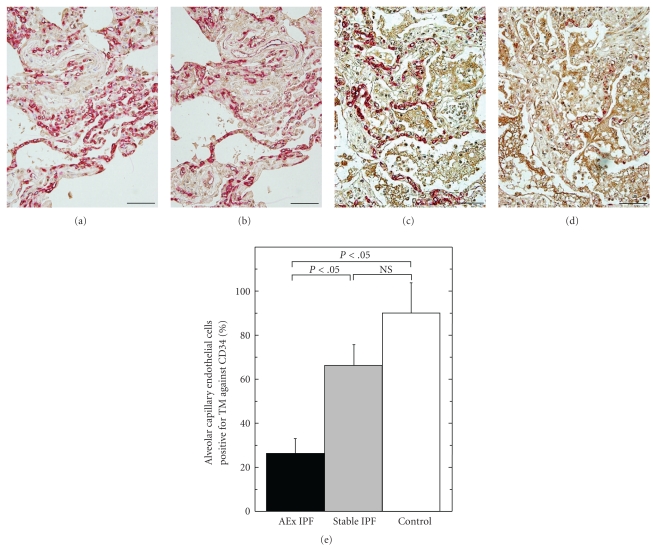
Distribution of alveolar capillary endothelial cells positive for thrombomodulin in stable IPF, acute exacerbation of IPF and controls. (a) and (b) The lung tissues which were surgically biopsied from a patient with stable IPF were double immunostained for HMGB1 (in brown) and CD34 (in red) (a). The consecutive sections were stained for HMGB1 (in brown) and thrombomodulin (in red) (b) (bars = 100 *μ*m). (c) and (d) The lung tissues which were obtained by autopsy from a patient who died after acute exacerbation were double immunostained for HMGB1 (in brown) and CD34 (in red) (c) or thrombomodulin (in red) (d) in the consecutive sections (bars = 100 *μ*m). (e) The percentages of the distribution areas of the capillary endothelial cells immunoreactive for thrombomodulin per those immunoreactive for CD34 were evaluated by a digital image analyzer and compared among stable IPF (*n* = 10), acute exacerbation (AEx IPF, *n* = 8) of IPF, and controls (*n* = 3).

The alveolar capillaries which were immunoreactive for CD34 expression (Figure 5(a)) and thrombomodulin (Figure 5(b)) were revealed in consecutive sections of a typical case of surgical biopsies. The immunoreactivity of thrombomodulin was almost always sustained in CD34-positive alveolar capillaries. In contrast, the endothelial expression of thrombomodulin was apparently decreased in the autopsied lung tissues of IPF patients after acute exacerbation (Figures 5(c) and 5(d)). Morphometric analysis using a digital image analyzer system clearly revealed the decrease of immunoreactivity for thrombomodulin compared to CD34 in the autopsied lung tissues of IPF patients died after acute exacerbation (26.1 ± 7.2%, *n* = 8) in comparison with controls (89.7 ± 13.8%, *n* = 3) and even with the biopsied lung tissues from stable IPF patients (66.5 ± 9.0%, *n* = 10). No significant difference was found between the stable IPF patients and normal controls in terms of thrombomodulin reactivity against CD34 (Figure 5(e)). 

## 4. Discussion

This is the first report of a persistent elevation of HMGB1 and MCP-1 after the onset of acute exacerbation in the lungs of patients with IPF. Both of these mediators were produced by alveolar macrophages and alveolar type II epithelial cells distributed in the capillary-increased alveolar lesions in which the capillary endothelial cells exhibited reduced expression of thrombomodulin, an intrinsic antagonist of HMGB1 [18].

The gradual increase of HMGB1 in the bronchoalveolar lavage fluid after the onset of acute exacerbation suggests several plausible roles in the pathogenesis of acute exacerbation of IPF. One of the roles of HMGB1 is as a late mediator of acute exacerbation, because the concentration of HMGB1 was low at the initial onset of acute exacerbation. Although no virulent organisms or viruses were detected in the bronchoalveolar lavage fluids examined in this study, all of these patients had flu-like symptoms a few days before the onset of acute exacerbation, which can cause a trigger-like effect of fulminating inflammation similar to acute lung injury. The gradual increase of HMGB1 in the epithelial lining fluids in the lungs after the initial onset of lung injury was also reported in both experimental and clinical acute lung injury [6]. Although HMGB1 can be released by macrophages in response to viral infection [23], the gradual increase of HMGB1 observed in this study is considered to be induced by passive release from necrotic alveolar type II epithelial cells [5] and by active secretion from alveolar macrophages in response to TNF*α*, IL-1, IFN*γ*, and oxidative stress without proved molecular mechanisms [24–26]. This is because HMGB1 increased in the absence of virulent virus and because the cytoplasmic staining of HMGB1 was significantly increased in these cells after acute exacerbation.

In contrast to the role promoting severe inflammation, HMGB1 has been reported to promote tissue repair and regeneration [27]. Importantly, HMGB1 induces the migration of stem cells toward inflamed regions to promote repair and regeneration [28]. For example, in smooth muscle cells, HMGB1 induces proliferation and rapid changes in cellular architecture, resulting in cell migration [29, 30]. Interestingly, many of these restorative effects are mediated through the same receptors (e.g., RAGE) that mediate the proinflammatory properties of the molecule [29]. Considering a recent report that the loss of RAGE contributes to pulmonary fibrosis [31], the increase of HMGB1 in alveolar fluids itself may induce fibrogenesis through other HMGB1 receptors, such as the Toll-like family of receptors (TLRs), TLR4, TLR2, or TLR9 [32, 33]. The gradual increase of MCP-1 in positive correlation with HMGB1 in alveolar fluids after acute exacerbation may also contribute to fibroproliferation [34, 35], which is thought to be involved in the regenerative effects of HMGB1. Of course, we cannot deny at this point the plausibility that treatment, including steroid therapy, for these patients in the hospital after the onset of acute exacerbation might affect the gradual increase of HMGB1. In addition, the correlation of the concentration of HMGB1 and/or MCP-1 in BALF with the disease severity of acute exacerbation of IPF has been left for expectations. 

We observed that the cells with cytoplasmic expression of HMGB1 were distributed in the alveolar capillary augmented lesions [15] in which the endothelial cells displayed reduced expression of thrombomodulin, an intrinsic antagonist of HMGB1 [18], after acute exacerbation. As we reported previously, even though neutrophils could be origin of HMGB1 in acute lung injury [7], in addition to alveolar epithelial cells and alveolar macrophages, the concentration of HMGB1 beside capillaries should be elevated. These capillary augmented lesions in IPF patients are susceptible to acute lung injury, not only because these capillaries reduce the ability to antagonize HMGB1 but also because VEGF, which is elevated (augmented) in these lesions [15], loosens the tight junctions of endothelial cells so as to induce leakage [36, 37]. The decreased expression of thrombomodulin, which is an anticoagulant [38] as well, may readily induce coagulation in these capillary augmented lesions, which frequently occurs in cases of acute exacerbation in IPF patients [39].

We conclude from these results that HMGB1 and MCP-1 are increased in the lungs of IPF patients after acute exacerbation and that the alveolar capillary augmented lesions with decreased expression of thrombomodulin, an intrinsic inhibitor of HMGB1, may exacerbate alveolar damage and fibrogenesis. 

## References

[1] Travis WD, King TE, Bateman ED (2002). American thoracic society/European respiratory society international multidisciplinary consensus classification of the idiopathic interstitial pneumonias. *American Journal of Respiratory and Critical Care Medicine*.

[2] Collard HR, Moore BB, Flaherty KR (2007). Acute exacerbations of idiopathic pulmonary fibrosis. *American Journal of Respiratory and Critical Care Medicine*.

[3] Klune JR, Dhupar R, Cardinal J, Billiar TR, Tsung A (2008). HMGB1: endogenous danger signaling. *Molecular Medicine*.

[4] Wang H, Bloom O, Zhang M (1999). HMG-1 as a late mediator of endotoxin lethality in mice. *Science*.

[5] Scaffidi P, Misteli T, Bianchi ME (2002). Release of chromatin protein HMGB1 by necrotic cells triggers inflammation. *Nature*.

[6] Ueno H, Matsuda T, Hashimoto S (2004). Contributions of high mobility group box protein in experimental and clinical acute lung injury. *American Journal of Respiratory and Critical Care Medicine*.

[7] Ogawa EN, Ishizaka A, Tasaka S (2006). Contribution of high-mobility group box-1 to the development of ventilator-induced lung injury. *American Journal of Respiratory and Critical Care Medicine*.

[8] Li J, Kokkola R, Tabibzadeh S (2003). Structural basis for the proinflammatory cytokine activity of high mobility group box 1. *Molecular Medicine*.

[9] Messmer D, Yang H, Telusma G (2004). High mobility group box protein 1: an endogenous signal for dendritic cell maturation and Th1 polarization. *Journal of Immunology*.

[10] Lotze MT, Tracey KJ (2005). High-mobility group box 1 protein (HMGB1): nuclear weapon in the immune arsenal. *Nature Reviews Immunology*.

[11] Abraham E, Arcaroli J, Carmody A, Wang H, Tracey KJ (2000). Cutting edge: HMG-1 as a mediator of acute lung inflammation. *Journal of Immunology*.

[12] Hori O, Brett J, Slattery T (1995). The receptor for advanced glycation end products (RAGE) is a cellular binding site for amphoterin. Mediation of neurite outgrowth and co-expression of RAGE and amphoterin in the developing nervous system. *The Journal of Biological Chemistry*.

[13] Palumbo R, Galvez BG, Pusterla T (2007). Cells migrating to sites of tissue damage in response to the danger signal HMGB1 require NF-*κ*B activation. *Journal of Cell Biology*.

[14] Park JS, Arcaroli J, Yum HK (2003). Activation of gene expression in human neutrophils by high mobility group box 1 protein. *American Journal of Physiology*.

[15] Ebina M, Shimizukawa M, Shibata N (2004). Heterogeneous increase in CD34-positive alveolar capillaries in idiopathic pulmonary fibrosis. *American Journal of Respiratory and Critical Care Medicine*.

[16] Fischer S, Gerriets T, Wessels C (2007). Extracellular RNA mediates endothelial-cell permeability via vascular endothelial growth factor. *Blood*.

[17] Mura M, Dos Santos CC, Stewart D, Liu M (2004). Vascular endothelial growth factor and related molecules in acute lung injury. *Journal of Applied Physiology*.

[18] Abeyama K, Stern DM, Ito Y (2005). The N-terminal domain of thrombomodulin sequesters high-mobility group-B1 protein, a novel antiinflammatory mechanism. *Journal of Clinical Investigation*.

[19] Taniguchi H, Ebina M, Kondoh Y (2010). Pirfenidone in idiopathic pulmonary fibrosis. *European Respiratory Journal*.

[20] Yamada S, Inoue K, Yakabe K, Imaizumi H, Maruyama I (2003). High mobility group protein 1 (HMGB1) quantified by ELISA with a monoclonal antibody that does not cross-react with HMGB2. *Clinical Chemistry*.

[21] Numazaki Y, Oshima T, Ohmi A (1987). A microplate method for isolation of viruses from infants and children with acute respiratory infections. *Microbiology and Immunology*.

[22] Suzuki A, Watanabe O, Okamoto M (2005). Detection of human metapneumovirus from children with acute otitis media. *Pediatric Infectious Disease Journal*.

[23] Jiang W, Bell CW, Pisetsky DS (2007). The relationship between apoptosis and high-mobility group protein 1 release from murine macrophages stimulated with lipopolysaccharide or polyinosinic-polycytidylic acid. *Journal of Immunology*.

[24] Rendon-Mitchell B, Ochani M, Li J (2003). IFN-*γ* induces high mobility group box 1 protein release partly through a TNF-dependent mechanism. *Journal of Immunology*.

[25] Rouhiainen A, Kuja-Panula J, Wilkman E (2004). Regulation of monocyte migration by amphoterin (HMGB1). *Blood*.

[26] Tang D, Shi Y, Kang R (2007). Hydrogen peroxide stimulates macrophages and monocytes to actively release HMGB1. *Journal of Leukocyte Biology*.

[27] Bianchi ME, Manfredi AA (2007). High-mobility group box 1 (HMGB1) protein at the crossroads between innate and adaptive immunity. *Immunological Reviews*.

[28] Palumbo R, Bianchi ME (2004). High mobility group box 1 protein, a cue for stem cell recruitment. *Biochemical Pharmacology*.

[29] Degryse B, Bonaldi T, Scaffidi P (2001). The high mobility group (HMG) boxes of the nuclear protein HMG1 induce chemotaxis and cytoskeleton reorganization in rat smooth muscle cells. *Journal of Cell Biology*.

[30] Porto A, Palumbo R, Pieroni M (2006). Smooth muscle cells in human atherosclerotic plaques secrete and proliferate in response to high mobility group box 1 protein. *The FASEB Journal*.

[31] Englert JM, Hanford LE, Kaminski N (2008). A role for the receptor for advanced glycation end products in idiopathic pulmonary fibrosis. *American Journal of Pathology*.

[32] Park JS, Gamboni-Robertson F, He Q (2006). High mobility group box 1 protein interacts with multiple Toll-like receptors. *American Journal of Physiology*.

[33] Tian J, Avalos AM, Mao SY (2007). Toll-like receptor 9-dependent activation by DNA-containing immune complexes is mediated by HMGB1 and RAGE. *Nature Immunology*.

[34] Moore BB, Peters-Golden M, Christensen PJ (2003). Alveolar epithelial cell inhibition of fibroblast proliferation is regulated by MCP-1/CCR2 and mediated by PGE. *American Journal of Physiology*.

[35] Inoshima I, Kuwano K, Hamada N (2004). Anti-monocyte chemoattractant protein-1 gene therapy attenuates pulmonary fibrosis in mice. *American Journal of Physiology*.

[36] Fischer S, Gerriets T, Wessels C (2007). Extracellular RNA mediates endothelial-cell permeability via vascular endothelial growth factor. *Blood*.

[37] Mura M, dos Santos CC, Stewart D, Liu M (2004). Vascular endothelial growth factor and related molecules in acute lung injury. *Journal of Applied Physiology*.

[38] Esmon CT (1989). The roles of protein C and thrombomodulin in the regulation of blood coagulation. *The Journal of Biological Chemistry*.

[39] Kubo H, Nakayama K, Yanai M (2005). Anticoagulant therapy for idiopathic pulmonary fibrosis. *Chest*.

